# Rare Paradoxical Response of Tachyarrhythmia to Adenosine Complicated by Novel ECG Artifact

**DOI:** 10.7759/cureus.31827

**Published:** 2022-11-23

**Authors:** Nathaniel A Carman, Mark E Peele, Alexandra J Smith

**Affiliations:** 1 Cardiology, Brooke Army Medical Center, San Antonio, USA

**Keywords:** supraventricular tachycardia (svt), wireless acquisition module, aberrancy, atrial flutter, artifact, adenosine

## Abstract

Adenosine is widely used for the diagnosis and treatment of supraventricular tachyarrhythmia. We report a rare case of adenosine use associated with the development of 1:1 atrial flutter with aberrancy. The diagnosis was further complicated by a newly described ECG artifact associated with Wireless Acquisition Module (WAMTM) ECG acquisition mimicking rhythm irregularity.

## Introduction

Supraventricular tachycardia (SVT) is a commonly encountered condition in emergency departments. SVT is used as an umbrella term to describe the ECG of several specific pathologic entities that can result in regular, narrow-complex tachycardia. While each of these underlying causes of SVT have their own nuances in long-term and definitive management, acute management is algorithmic and described in published joint societal guidelines. Intravenous adenosine carries a class I recommendation as one of the first interventions for acute SVT because of its efficacy and rare serious adverse effects [1]. Here, we present a case of SVT with an exceptionally rare paradoxical response to intravenous adenosine.

## Case presentation

A 26-year-old woman experienced sudden onset of palpitations and rapid heart rate immediately after sipping a frozen margarita and experiencing a cold stimulus headache or “brain freeze”. She is a healthcare worker and noted her smart watch recording a pulse of 150 beats per minute. She self-initiated vagal maneuvers with no resolution. She presented via emergency medical services to the emergency department and upon arrival had blood pressure 156/94 mmHg, heart rate 148 beats/minute, respirations 18/min, and oxygen saturation 99% on room air. Physical examination revealed tachycardia, regular rhythm, no rubs, murmurs, or gallops, clear lung sounds, and no peripheral edema. The patient had no past cardiac history and was taking no medications.

**Figure 1:**
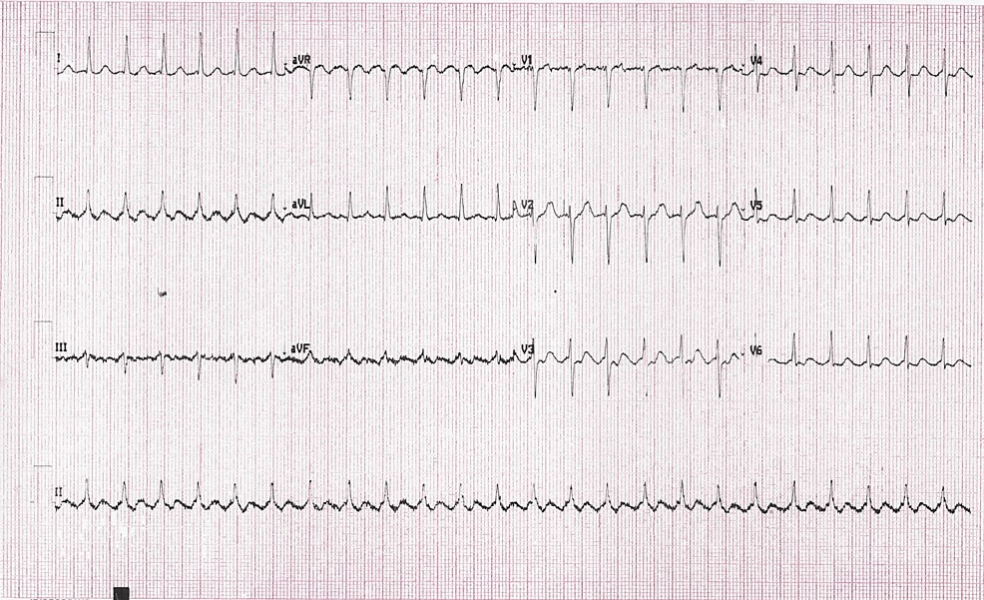
ECG on presentation to the emergency department.

The initial differential diagnosis included tachycardia from physiologic stress, hyperthyroidism, panic, anxiety, and cardiac dysrhythmia including atrial fibrillation (AF), atrial flutter (AFL), SVT, and ventricular tachycardia. The initial electrocardiogram (Figure 1) showed regular, narrow-complex tachycardia at 147 beats/min. Laboratory examination revealed a troponin-T of <0.010 ng/ml. Serum electrolytes and thyroid studies were within normal range and a urine drug screen was negative.

**Figure 2:**
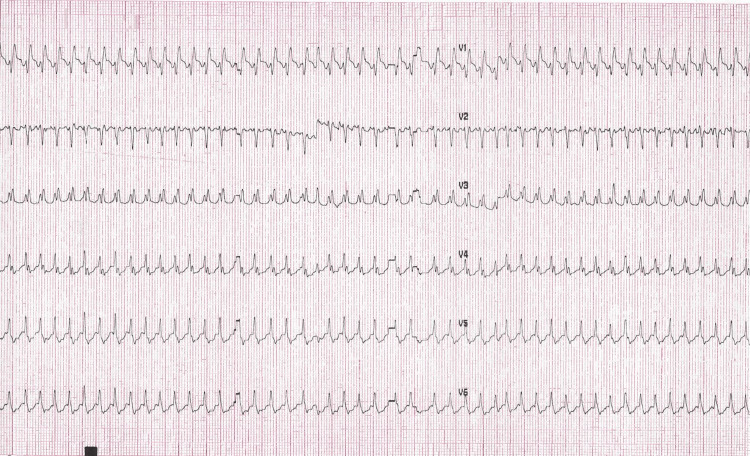
Rhythm strip immediately following adenosine 6 mg intravenous push.

**Figure 3:**
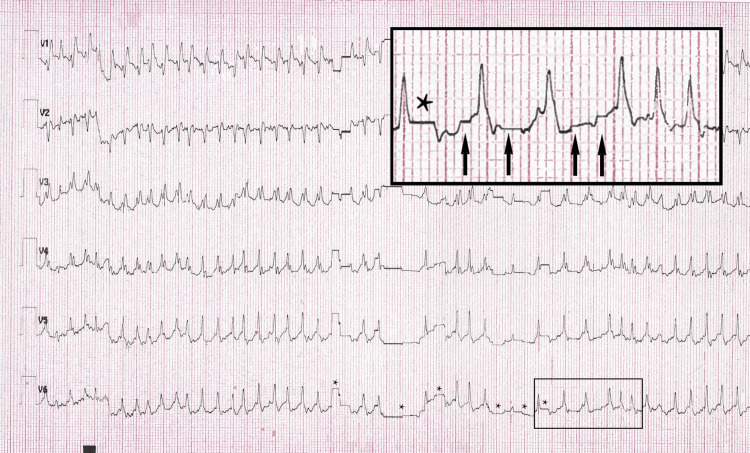
Rhythm strip immediately following intravenous push of adenosine 6 mg demonstrating artifact mimicking rhythm irregularity as well as subtle variation in QRS morphology. Asterisks (*) in lead V6 highlight some more noticeable examples of artifact. The inlaid image demonstrates close-up view highlighting more subtle artifacts (arrows) and further demonstrating artifact contribution to variation in QRS morphology.

**Figure 4:**
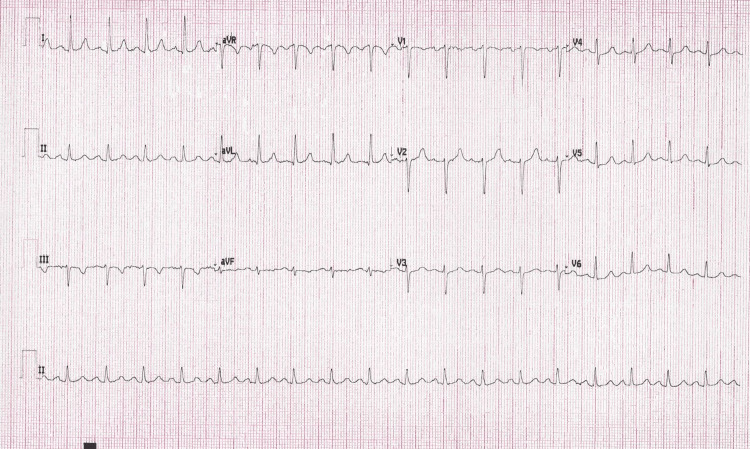
ECG status post-synchronized cardioversion demonstrating sinus tachycardia.

In the emergency department, an initial diagnosis of SVT versus 2:1 AFL was made. In line with current SVT guidelines, vagal maneuvers were attempted with a reported increase in heart rate to 300 beats/min [1]. This was followed by a rapid intravenous push of 6 mg adenosine which resulted in sustained rhythm change to a wide-complex tachycardia at approximately 300 beats/min with periods of irregularity (Figures 2-3). The patient reported lightheadedness and the decision was made to perform electrical cardioversion for hemodynamic instability. The patient was emergently sedated with etomidate 20 mg intravenously and underwent synchronized cardioversion at 100 joules with the successful restoration of sinus rhythm. Repeat ECG demonstrated sinus tachycardia with normal intervals and no evidence of pre-excitation (Figure 4). The patient was admitted to the cardiology service and a diagnosis of AFL was made. Adenosine administration was deemed paradoxically associated with conversion to 1:1 AFL with a change in QRS morphology attributable to incomplete right bundle branch block aberrancy. A transthoracic echocardiogram was obtained revealing normal chamber sizes, wall thickness, and systolic function with no significant valve disease. The patient was started on oral beta-blocker therapy and discharged with no evidence of recurrent arrhythmia for the duration of hospitalization. After shared decision-making, anticoagulation was deferred given her CHA2DS2-VASc score of 1 (female sex). Electrophysiology study and ablation were deferred after shared decision-making with a plan to pursue further intervention should arrhythmia recur.

## Discussion

This case presented multiple diagnostic difficulties including AFL presenting in an uncommon patient demographic, rare paradoxical response to adenosine, and morphological change in QRS with the perception of irregularity on ECG. These confounders initially clouded the diagnosis between AFL, SVT, pre-excited AF, and ventricular tachycardia.

AFL in the young is uncommon and far less common than SVT in this demographic [2,3]. Among children and adults diagnosed with AFL, the vast majority of cases are associated with other congenital cardiac diseases, with only 8% of patients in a large observational study found to have an otherwise normal heart [4].

Adenosine use is associated with multiple adverse reactions but these are typically short-lived and rarely serious making its use generally considered safe [1,5,6]. Although previously described in the literature, it is exceptionally rare for adenosine administration to result in enhanced atrioventricular (AV) nodal conduction and provocation of 1:1 AFL [7-9]. Four total cases of this phenomenon were identified in our literature search, including one in a 12-year-old with congenital heart disease and three in adults between 53 and 67 years old. In two cases, the patient suffered cardiac arrest associated with 1:1 AFL requiring cardioversion/defibrillation. It has been previously postulated that this paradoxical enhanced AV nodal conduction may be secondary to increased activation of the sympathetic nervous system [10].

The diagnosis was further complicated by the development of rate-related aberrancy with conversion to 1:1 AFL further broadening the differential to include causes of wide-complex tachycardia.

Further confounding the diagnosis is an ECG artifact that is newly described and reported here. This artifact, demonstrated best in Figure 3, gives the appearance of substantial irregularity. While obvious in some areas of the tracing, marked with asterisks (*), it is subtle in others. We highlight the more subtle examples with arrows in the inlaid and enlarged section where the artifact can be better appreciated to also result in significant QRS morphology variation. This tracing could be misinterpreted as the development of a wide-complex, irregular tachycardia after adenosine administration, which raises concern for pre-excited AF with a very short cycle length and potential for deterioration into ventricular fibrillation. While the patient’s sinus rhythm ECG demonstrated no evidence of pre-excitation (Figure 4), rapidly conducting accessory pathways that only exhibit intermittent pre-excitation at rest have been described in patients in high sympathetic output states [11]. In consultation with industry representatives, we determined that this artifact was likely secondary to a low double AA battery in the Wireless Acquisition Module (WAM)TM (Welch Allyn, Inc., Skaneateles Falls, NY) used in this case with the ELITM 380 Electrocardiograph (Hillrom, Chicago, IL) (personal communication). In our review, this artifact has not been described before in the medical literature.

## Conclusions

This case describes an exceptionally rare response to adenosine and underscores appropriate caution and the importance of readily available resuscitative equipment when using this drug. We highlight features complicating diagnosis in this case with emphasis on recognizing an ECG artifact that may become more commonly encountered with routine use of wireless ECG acquisition technology.
